# Water-Mediated Conversion of BaTiO_3_ Nanoparticles into BaCO_3_ Nanorods in Electrospun Polymer Fibers: Implications for Carbon Capture Applications

**DOI:** 10.1021/acsanm.3c03703

**Published:** 2023-10-19

**Authors:** Hasan Razouq, Kerstin Neuhauser, Gregor Zickler, Thomas Berger, Oliver Diwald

**Affiliations:** Department of Chemistry and Physics of Materials, Paris-Lodron University Salzburg, Jakob-Haringer-Straße 2a, A-5020 Salzburg, Austria

**Keywords:** BaTiO_3_ nanoparticles, barium ion leaching, gas phase synthesis, nanoparticle carbonation, material processing, chemical weathering, electrospinning

## Abstract

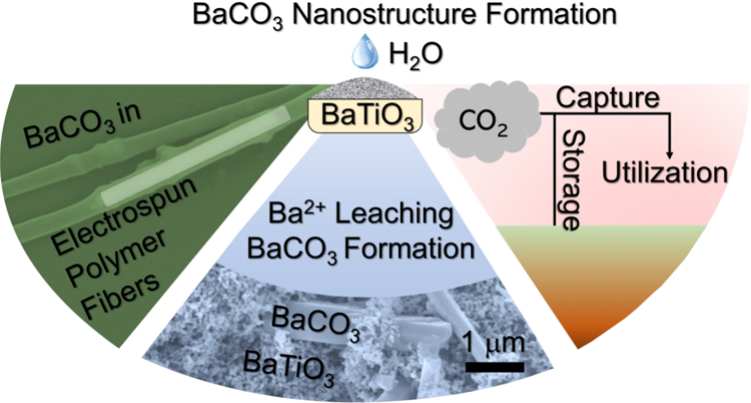

Under ambient conditions and in aqueous environments, transformations of nanoparticle-based ferroelectric components can raise important stability issues that are relevant for applications as multilayer capacitors, flexible piezoelectrics, or biomedical devices. We show that X-ray amorphous BaTiO_3_ nanoparticles that were grown by flame spray pyrolysis and which can be incorporated into electrospun polymer fibers undergo incongruent Ba^2+^ dissolution in the presence of water. At pH > 5 and in contact with air, corresponding Ba solutes spontaneously convert into crystalline BaCO_3_ needles to produce characteristic nano- and microstructures. We compared the reactivity of amorphous BaTiO_3_ nanoparticle powders with those of nanocrystals after annealing-induced crystallization. The stability of aqueous nanoparticle–polymer formulations, which are typically part of nanoparticle encapsulation in polymers and electrospinning, was included in this analysis. Nanoparticle size, crystallinity, surface area, the presence of carbonaceous surface contaminants, and the effect of surface passivation with polymers are addressed to underline the critical role of condensed water during the synthesis, storage, and processing of BaTiO_3_ nanoparticle-based composites.

## Introduction

The stability of metal oxide nanostructures in aqueous environments is a key factor in the synthesis and processing of nanomaterials. Moreover, the understanding of the size-dependent stability of particle powders in contact with liquid water and/or CO_2_ is very important for chemical weathering or aging of mineral binders.^1−3^ In particular, the reactivity of metal oxides of enhanced basicity, such as alkaline earth oxides, toward CO_2_ has been addressed and the concomitant formation of carbonates with their characteristic crystal growth modes is of interest in the field of carbon capture and storage (CCS).^4^

BaCO_3_ adopts characteristic crystal habits, such as rod-, whisker-, or needle-like crystal shape. There exists a wealth of information about BaCO_3_ formation in aqueous environments. Great advances have been made about tuning the crystallite growth and morphology.^5,6^ The utilization of surface chemistry involving additives^7^ such as zwitterions, polymers, and/or polyelectrolytes has led to appropriate routes toward advanced mesocrystal architectures. Approaches for the generation of helical BaCO_3_ mesocrystals^8,9^ that are built up from faceted and elongated BaCO_3_ nanocrystals have been reported recently.

Perovskite BaTiO_3_ nanoparticles, on the other hand, have gained substantial interest due to their ferroelectric properties and the compound’s high dielectric constant.^10,11^ Apart from their importance as source materials for the production of electroceramics, the size and scaling effects were studied as a result of the continuous miniaturization of electronic components, such as multilayer ceramic capacitors (MLCCs).^12^ Polymer-based nanocomposite films that contain BaTiO_3_ nanoparticles show favorable dielectric properties, while maintaining the transparency, flexibility, and workability of the polymer films.^13^ The acceptable biocompatibility of BaTiO_3_ nanoparticles makes them promising candidates for biomedical applications and wearable bioelectronics.^11^

The solid-state synthesis of BaTiO_3_ (BTO) typically involves polycrystalline BaCO_3_ and TiO_2_ powders as educts.^14^ In the opposite direction, however, BTO can convert into BaCO_3_^15^ in aqueous environment which is linked to technologically important processing routes of this compound.^15−18^ This topic is particularly relevant for the utilization of submicrometer-sized BTO materials that need to be formulated for shaping of functional ceramics, such as the abovementioned MLCCs or piezoelectric devices. Manufacturing of such materials inherently involves colloidal processing in aqueous media, where related nanoparticle dispersions are cast, laminated, and sintered.

**Scheme 1:**
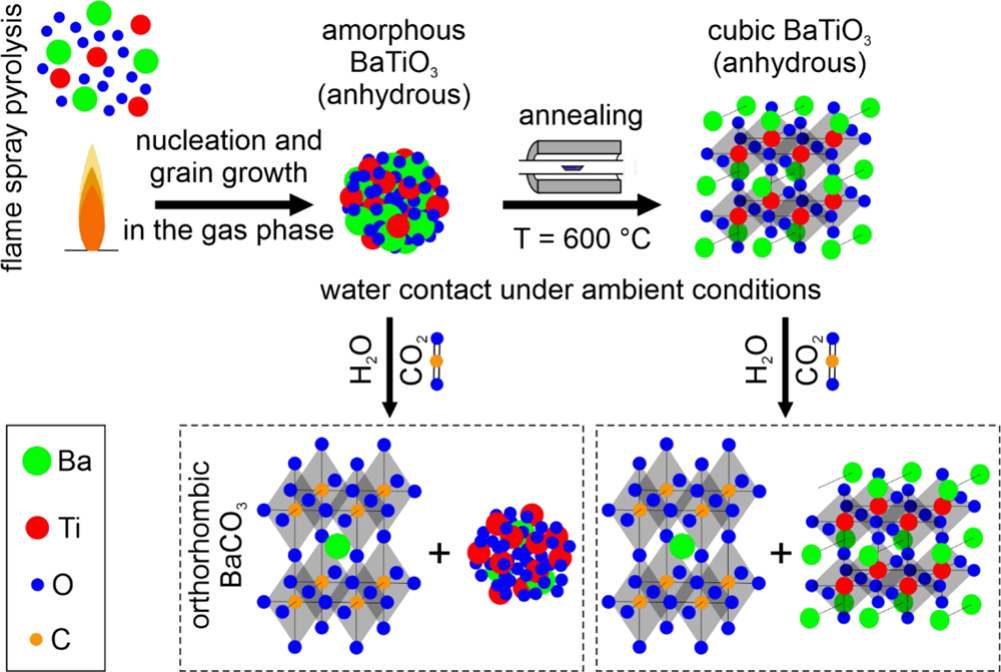
Systematic Presentation of Experiments That Were Performed To Explain BaTiO_3_ (BTO) Nanoparticle Stability during Processing and Electrospinning in Aqueous Poly(vinyl alcohol) (PVA) Formulations

Here, we report on unexpected compositional and morphological transformations of gas-phase synthesized BTO nanoparticles in aqueous nanoparticle/poly(vinyl alcohol) dispersions upon colloidal processing and electrospinning. The fabrication of continuous fibers with diameters that can be scaled down to a few nanometers^19^ is particularly well-suited for the production of composite nanofibers incorporating metal oxide nanoparticles. These composite fibers give rise to significant specific surface areas of the material which hosts a rich network of particle–particle interfaces, enhancing their performance as sensors or catalyst components.^20^ Poly(vinyl alcohol) (PVA), a widely used additive and polymer, offers several advantageous properties: it is highly hydrophilic, nontoxic, and biocompatible.^21^ Loaded with BTO nanoparticles, such ultrafine fibers can exploit the properties of the insulating, semiconducting,^22^ or piezoelectric properties that are used for flexible nanogenerators.^23^ Motivated by the abovementioned unexplained materials transformations inside the polymer matrix, we carefully evaluated the process chain starting with amorphous BaTiO_3_ nanoparticles and their interaction with water (Scheme 1) and succeeded in explaining the phenomena in terms of composition and structure of the particles (Scheme 1).

This study is structured as follows: first, we report on the structural and compositional properties of electrospun nanoparticle/polymer fibers and address the water-mediated conversion of BTO nanoparticles into BaCO_3_ rods in detail. Finally, we discuss the implications of these findings for colloidal processing of metal oxide nanomaterials for functional ceramics,^10,12^ BTO-polymer nanocomposites,^13,24−26^ or the potential of Ba^2+^ leaching for CO_2_ adsorption.^4,27^

## Experimental Methods

### Particle Synthesis

We produced the barium titanate nanoparticle powder with a flame spray pyrolysis approach (FSP). The apparatus used consists of a syringe pump, a burner, and a powder-collecting unit.^28,29^ The syringe pump (LA-160, HLL Landgraf) supplied the liquid fuel-precursor mixture at a constant flow rate of 2 mL/min. The precursor is transferred to the second unit (the burner) and sprayed through a nozzle. Oxygen dispersion gas (O_2_ 5.0, dispersion gas) converts the precursor feed into fine droplets. The dispersion gas is supplied at a volume flow of 3.0 L/min through a surrounding annular gap. The precursor fine droplets are ignited by a concentrically arranged methane and oxygen combustion flame (CH_4_ 4.5, 1.5 L/min, O_2_ 5.0, 2.0 L/min). Oxygen sheath flow (sheath gas, O_2_ 5.0, 5.0 L/min) is guided through a sintered metal plate ring to ensure stoichiometric oxides production and to avoid byproducts. Mass flow controllers (Bronkhorst EL-FLOW) guarantee constant gas flow rates during the synthesis. A vacuum pump (Busch Seco SV 1040 C) ensures particle flow toward the particle collection unit. This unit consists of a glass fiber filter (Hahnemühle, GF6, Ø 257 mm) located on a water-cooled filter holder. Synthesis-related production parameters used for the synthesis of the here investigated nanoparticle powders are listed in Table 1.

**Table 1:** Process Parameters for BTO Synthesis via Flame Spray Pyrolysis^30^

metal–organic precursor flow/mL min^–1^	2.0
dispersion gas (O_2_)/L min^–1^	3.0
supporting flame (CH_4_)/L min^–1^	1.5
supporting flame (O_2_)/L min^–1^	2.0
sheath gas (O_2_)/L min^–1^	5.0
pressure drop/bar	2–3

To prepare the precursor solution, the Ba precursor was dissolved under vigorous stirring at 120 °C for 3 h in 2-ethyl hexanoic acid. The liquid titanium precursor (titaniumisopropoxide, TTIP) is mixed with toluene and stirred for 3 h at room temperature before mixing with the dissolved Ba precursor.^30^ Corresponding mixing ratios for a 100 mL batch are exemplified in Table 2 and give rise to a Ti/Ba atomic ratio of 1:1.

**Table 2:** Precursor Parameters for the Synthesis of BTO via Flame Spray Pyrolysis^30,31^

metal–organic precursor	amount (g)	dissolved in	amount (mL)
titanisopropoxide (TTIP) Sigma-Aldrich 97%	4.83	toluene Sigma-Aldrich 99,8%	50
bariumacetate (BaAc) EMSURE ACS p.a.	4.34	2-ethylhexansäure Sigma-Aldrich ≥99%	50

### Annealing

After production, the BTO nanoparticle powder is transferred into fused silica cells, which allow thermal powder activation in alternating gas atmospheres of high vacuum (*p* < 10^–5^ mbar) or O_2_ atmosphere. Stepwise heating to 873 or 1173 K using a rate of 10 K min^–1^ in a high vacuum (*p* < 10^–5^ mbar) was performed to reduce surface contamination. After reaching the target temperature, the powder samples are exposed two times to molecular oxygen (20 mbar) for 30 min with a vacuum step for 30 min (*p* < 10^–5^ mbar) in between.^30^ This process removes carbon remnants arising from the metal–organic precursor by converting them into volatile CO and CO_2_, which are removed upon continuous pumping. Fresh oxygen is added during cooling to room temperature to ensure carbon removal and to prohibit defect formation. Powders resulting from vacuum annealing to 873 or 1173 K are designated as the VA873 sample and VA1173 sample, respectively. Powders that were not subjected to any post-synthesis treatment are designated as-synthesized samples.

### Electrospinning

Aqueous poly(vinyl alcohol) (PVA) (98.0–98.8% hydrolyzed M.W. 31,000–50,000) polymer solutions were used as a precursor for electrospinning, in which BTO nanoparticle powders were dispersed. Initially, a specific amount of polymer powder was dissolved in high-purity water (23 g/100 mL) under continuous stirring at 90 °C for 3 h, resulting in a concentration of 23% w/v. Then, a specific amount of nanoparticle powder (60 mg/mL) was added to the polymer solutions under vigorous stirring. The dispersion was subjected to 30 min of stirring followed by 15 min of ultrasonic treatment. The materials were electrospun (Starter Kit Random, Linari Nanotech) by applying a potential of 18 kV, a pumping rate of 0.05 mL/h, and a 20 cm distance between the needle tip and the collector.

### Electron Microscopy

Electron microscopy images were acquired by using scanning electron microscopy (SEM) and transmission electron microscopy (TEM). The SEM instrument (Zeiss FE-Ultra Plus 55) is equipped with a field-emission gun and Gemini lenses and was used at short working distances of around 3 mm and an accelerating voltage between 5–10 kV, with InLens and SE detectors. The TEM (JEOL JEM-F200 TEM) was operated at 200 kV and is equipped with a cold field emission electron source, a TVIPS F216 2k by 2k CMOS TEM camera, and a large windowless JEOL Centurio EDX detector. Particle size distribution plots before and after sintering and fiber diameters after thermal treatment were obtained from TEM images using the EM Measure software program from TVIPS. Selected area electron diffraction (SAED) images were recorded using a TVIPS F216 2k x 2k CMOS camera. The chemical composition of the samples is investigated by high-angle annular dark field (HAADF) images and energy-dispersive X-ray spectroscopy (EDX) maps.

### X-ray Diffraction (XRD)

X-ray diffraction data were collected at room temperature in coupled Theta–Theta mode on a Bruker D8 Advance diffractometer with a DaVinci design. The crystallite domain size was determined by applying the Scherrer equation to the main diffraction features.^32^ For quantitative phase analysis, the Rietveld method was applied by using the software TOPAS 4.2 (Bruker 2012). All X-ray diffraction measurements were performed on powder or ground samples to ensure a sufficient diffraction intensity that satisfies counting statistics. Electrospun fibers were frozen in liquid nitrogen for their solidification before being ground to form a powder.

### Thermogravimetric Analysis

Thermogravimetric analyses were performed using an STA 449 F3 Jupiter instrument from Netzsch. The measurements were carried out in the range from room temperature to 1273 K and with a heating rate of 10 K/min under synthetic air. To perform the analysis, the nanoparticle powder was placed on an aluminum oxide crucible, which served as a sample holder.

### Nitrogen Physisorption

Nitrogen physisorption was performed at 77 K (ASAP 2020 adsorption porosimeter, Micromeritics GmbH) and the specific surface area was calculated by applying the model of Brunauer–Emmet–Teller (BET). Prior to nitrogen sorption, the sample was degassed under a vacuum at 573 K for 3 h. The BET surface area was evaluated using adsorption data in a relative pressure range *p*/*p*_0_ of 0.06–0.21.

### Exposing the Powder to Condensed Water and in the Absence of Polymer

Approximately 50 mg of the nanoparticle powder was placed onto a Silicon wafer and then covered with a drop of deionized water (200 μL). Right after, the resulting mixture was dried in air (for *t* ≤ 30 min) at room temperature for further investigations. The samples investigated via SEM were dried at 80 °C to ensure complete water removal.

## Results and Discussion

**Figure 1:**
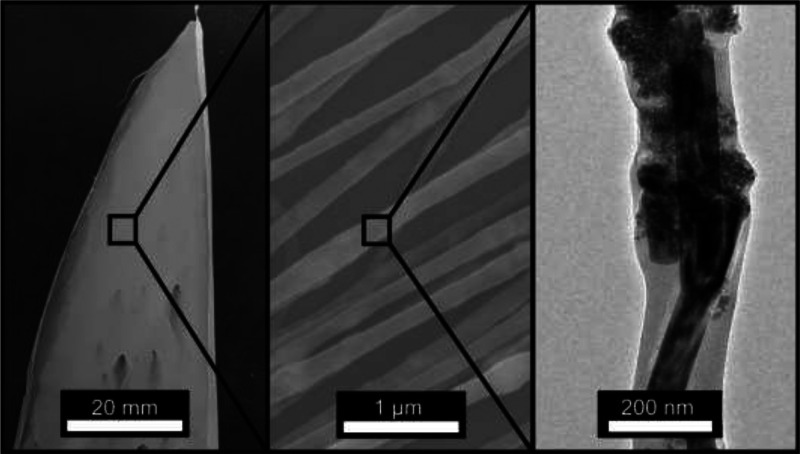
Digital photograph (left panel) and electron microscopy images—SEM image in the center and TEM image in the right panel—of electrospun PVA fibers from aqueous PVA formulations incorporating as-synthesized BTO nanoparticle powders.

The systematic evaluation of BTO nanoparticle powder reactivity toward liquid water was triggered by observations from polymer-encapsulated nanoparticles after their formulation with aqueous poly(vinyl alcohol) (PVA) mixtures for electrospinning (Figure 1).^20^ If highly dispersed vapor phase grown metal oxide nanoparticles, such as those of TiO_2_ or ZnO, show no reactivity toward water and the polymer, characteristic threads of particle chains can be isolated after annealing induced polymer removal.^20^ The microstructural situation of as-synthesized BTO nanoparticle powders in water formulated poly(vinyl alcohol) (PVA) dispersions, however, is very different (Figure 1).

At the length scale of micrometers (SEM image in the middle of Figure 1) the PVA-based fibers appear uniform, and the corresponding transmission electron microscopy (TEM) data (right panel of Figures 1 and S1) show encapsulated particles of high morphological definition which are much larger in size than the electrospun BTO nanoparticles forming agglomerates. Related rodlike features are elongated and clearly delimited by faceted interfaces. Apart from the fact that nanoparticle encapsulation inside electrospun fibers reveals important details about the organization of metal oxide particles inside the spatially confined organic matrices,^33^ such a material integration approach is also advantageous for the in-depth structural analysis of individual particles by electron diffraction. In fact, selected area electron diffraction (SAED) measurements on such rods yield diffraction spots consistent with the BaCO_3_ structure (Supporting Information, Figure S2).

**Figure 2:**
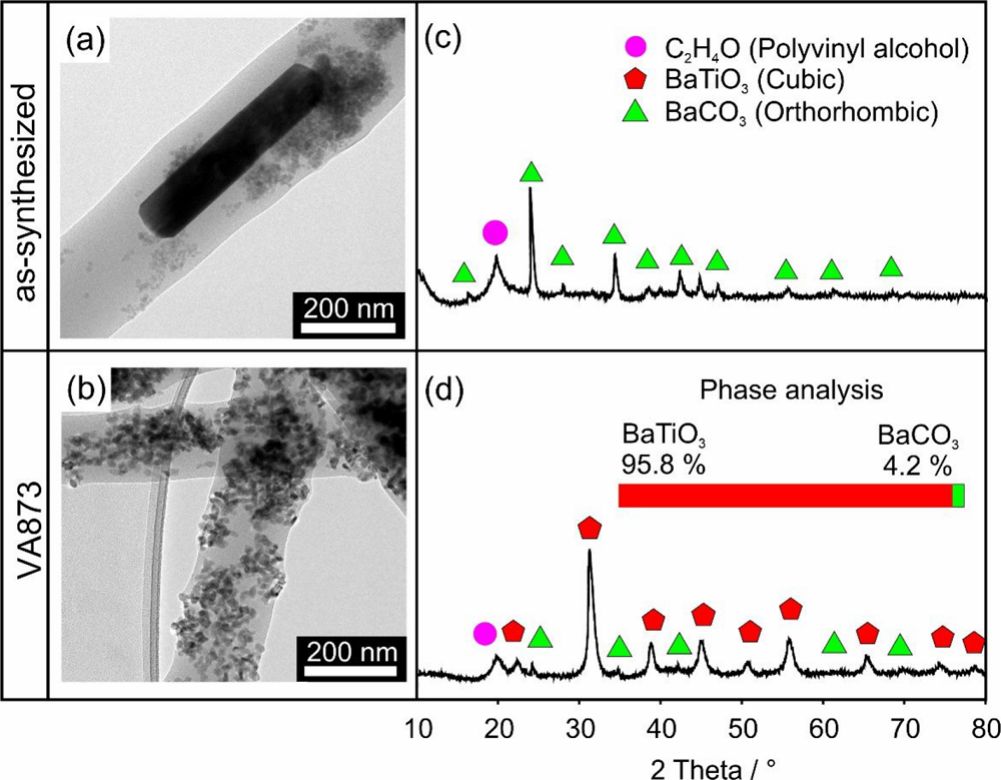
Characteristic TEM images (a, b) and XRD patterns (c, d) of electrospun fibers containing as-synthesized BTO nanoparticles (a, c) or BTO nanoparticles that were annealed to 873 K (VA873) prior to electrospinning (b, d). The inset in (d) highlights the abundance of the two crystalline phases, as determined by Rietveld refinement.

TEM and XRD data reveal significant differences for electrospun fibers depending on the thermal pretreatment of the inorganic particles prior to polymer encapsulation. When the as-synthesized powder was used as a precursor for the electrospun fibers, the electron micrograph (Figure 2a) indicates the presence of larger rod-shaped particles, which, in addition to the as-synthesized particles, were frequently observed in such samples and attributed to BaCO_3_. The diffraction pattern acquired on collected mats of such electrospun fibers (c) shows one PVA-specific reflection peak at 2Θ = 19° and a diffraction pattern that reveals BaCO_3_ as the only crystalline phase present (Figure 2c). In contrast, the electron micrographs of electrospun PVA fibers loaded with VA873 nanoparticle powders (Figure 2b) show uniformly sized fibers that exclusively host small nanoparticle agglomerates. As shown recently, thermal annealing to *T* ≥ 873 triggers the crystallization of amorphous BTO to convert into powders of BTO nanocrystals.^30^ Following electrospinning, TEM analysis of many different sample regions did not reveal any evidence of BaCO_3_ rods. In this case, XRD analysis confirms that the BTO nanoparticles adopt the tetragonal BTO phase and coexist with traces of crystalline BaCO_3_ (see weak diffraction features marked with green triangles, bottom of Figure 2d). A quantitative phase analysis of the electrospun fibers loaded with BTO VA873 nanoparticle powders using Rietveld refinement indicates that only 4% of the total crystalline material adopts the orthorhombic BaCO_3_ phase. Obviously, BTO VA873 exhibits enhanced stability toward BaCO_3_ formation in the presence of PVA. A significantly higher fraction of ∼ 14% of the total crystalline material was found to adopt the orthorhombic BaCO_3_ phase in polymer free aqueous dispersions (see below). As pointed out in a previous study,^34^ the dissolution properties of Ba^2+^ ions from BTO are significantly reduced by PVA in perfect agreement with our observations.

For the systematic analysis of the reactivity of FSP-derived nanoparticle powders toward water, we compared powder samples that were subjected to three different preannealing procedures. Specifically, samples of as-synthesized nanoparticle powders were vacuum-annealed to 873 K (BTO VA873) and 1173K (BTO VA1173), respectively (Table 3).

**Table 3:**
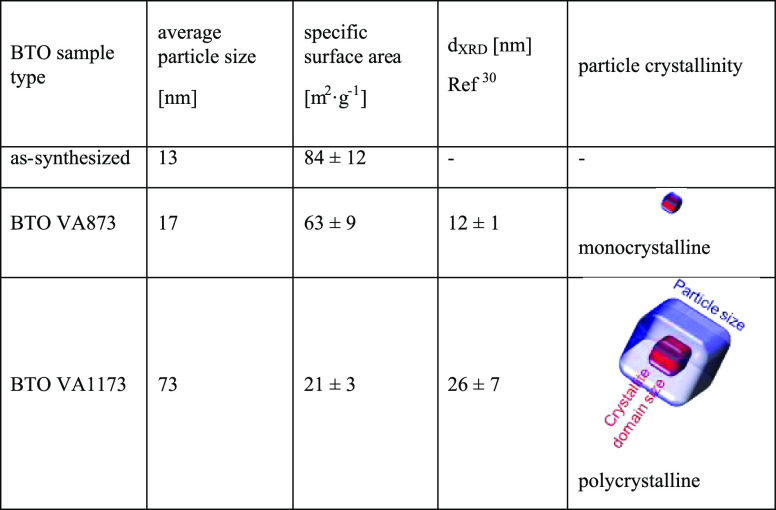
Comparison of the Particle Size, Crystallite Domain Size, and BET-Specific Surface Area Values of As-Synthesized BTO Nanoparticle Powders and BTO Nanoparticle Powder Annealed to 873 and 1173 K

**Figure 3:**
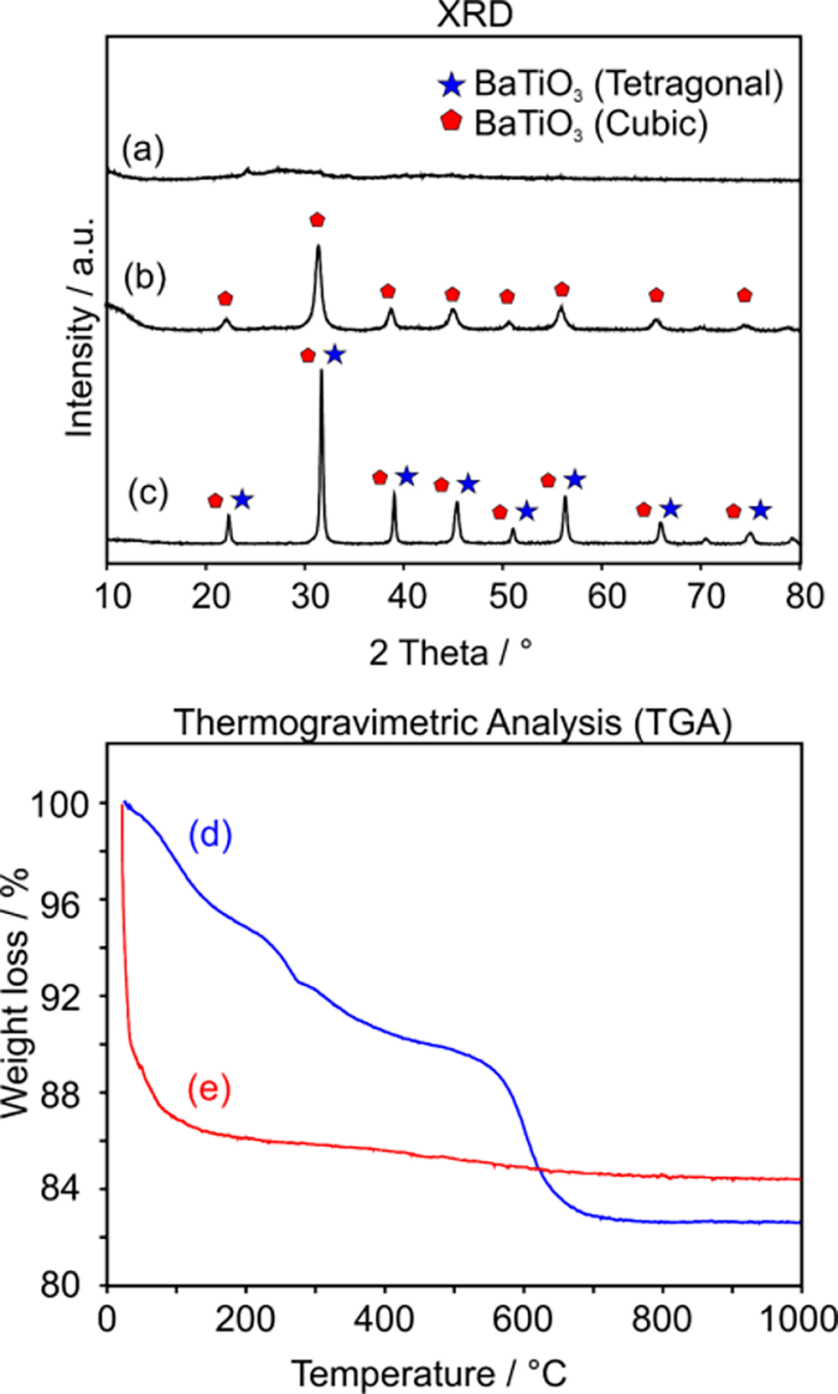
XRD patterns of as-synthesized BTO (a), BTO VA873 (b), and BTO VA1173 nanoparticle powders (c) and thermogravimetric analysis (TGA) results (d and e) obtained on as-synthesized BTO (d) and BTO VA873 nanoparticle powder (e) in synthetic air. Heating rate: 10 °C min^–1^.

XRD (Figure 3a–c) and thermogravimetric analysis (Figure 3d,e) as well as TEM analysis (including the particle size distribution functions derived from the TEM data Figure S3, Supporting Information) provide (i) structural and morphological information about annealing-induced particle crystallization (Figure 3a–c), (ii) the decomposition and elimination of synthesis-related carbon and water residues (Figure 3d,e), and (iii) particle coarsening (Figure S3, Supporting Information).

The as-synthesized nanoparticle powder is X-ray amorphous with no crystalline diffraction features observed (Figure 3a, Table 3). The vacuum-annealed powder BTO VA873 shows diffraction features that are consistent with the cubic BTO phase (Table 3). Evaluation of the diffraction signal width points to an average crystallite domain size of 12 ± 1 nm. The XRD pattern of BTO VA1173 (Figure 3c) reveals the presence of the cubic and the tetragonal BTO phase. (Due to broadened diffraction features and based on XRD measurements alone, it is impossible to provide in this case a quantitative phase analysis.) Earlier Raman spectroscopy measurements on comparable materials^30,35^ in fact confirmed that both BTO phases, cubic and tetragonal BTO, are present in the BTO VA873 and BTO VA1173 samples. The crystallite domain size was calculated to be around 26 ± 7 nm. Comparing this value with the average particle size (Table 3 and Figure S3f, Supporting Information) suggests that related particles are polycrystalline.^30^

Driven by surface and interface minimization, particle coarsening is induced by annealing treatment at temperatures *T* ≤ 873 K which triggers ion diffusion and mass transport in general. We expect that under the vacuum treatment applied here, particle coalescence contributes substantially to the growth of the grains.

As-synthesized nanoparticles are hydrated and contain synthesis-related carbon remnants. TGA of a pristine powder sample reveals that annealing to 873 K gives rise to a mass loss of about 17% (Figure 3d). Prior to their stability tests in water (as presented below), the application of 2–3 powder exposure cycles to oxygen (30 min each) with intermediate evacuation steps (continuous pumping to *p* < 10^–5^ mbar for 30 min) is sufficient to effectively remove carbon from the BTO VA873 (Figure 3e) and BTO VA1173 powder samples.

**Figure 4:**
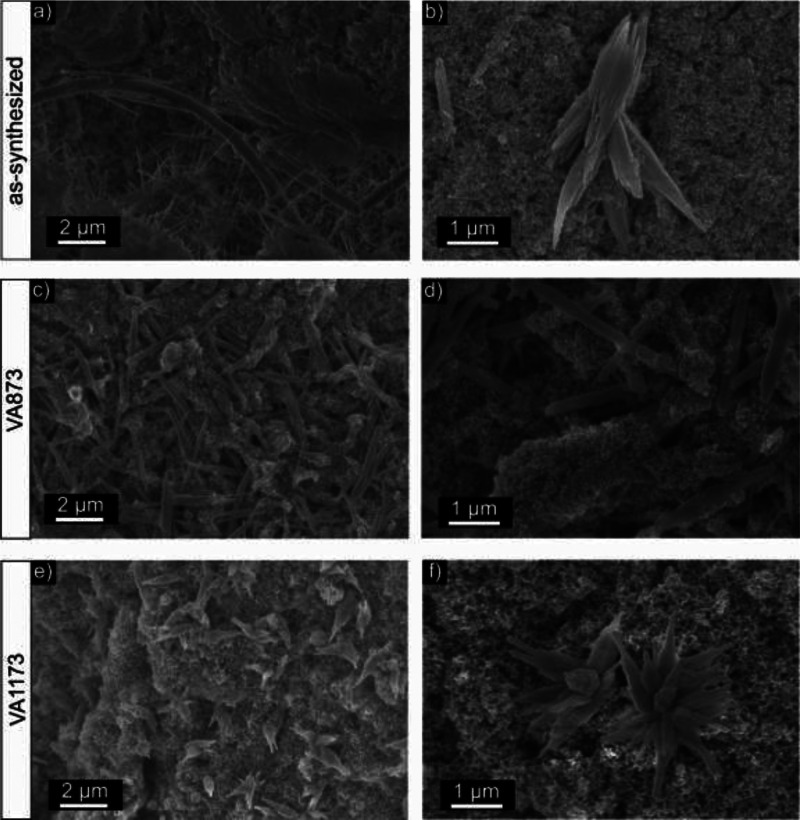
SEM images of as-synthesized BTO (a, b), BTO VA873 (c, d), and BTO VA1173 (e, f) nanoparticle powders after room temperature contact with liquid water and subsequent drying in air.

We exposed as-synthesized as well as annealed powder samples to liquid water in air and at room temperature. Approximately 50 mg of the nanoparticle powder was deposited on a silicon wafer and covered with a drop of deionized water with an approximate volume of 200 μL. Drying in air and for a time interval not longer than 30 min leads to samples as characterized by SEM in Figure 4.

Exposure of as-synthesized BTO powder to water produces elongated structures, including needle-like, rod-shaped, and hairlike crystallites that grow out of aggregates of spherical nanoparticles (Figure 4a,b). The elongated structures show a wide range of sizes, between tens of nanometers to micrometers. Similar elongated structures were also observed in the VA873 powder sample after water contact (Figure 4c,d). In this case, the size distribution of the rods with lengths of a few micrometers and their morphology seem to be more uniform and homogeneous, respectively. The SEM analysis of the VA1173 powder sample after water contact (Figure 4e,f) reveals clusters of roselike BaCO_3_ structures that have formed at specific positions of the nanoparticle-based substrate. In terms of morphology, these structures are more uniform as compared to the as-synthesized powder after water contact.

**Figure 5:**
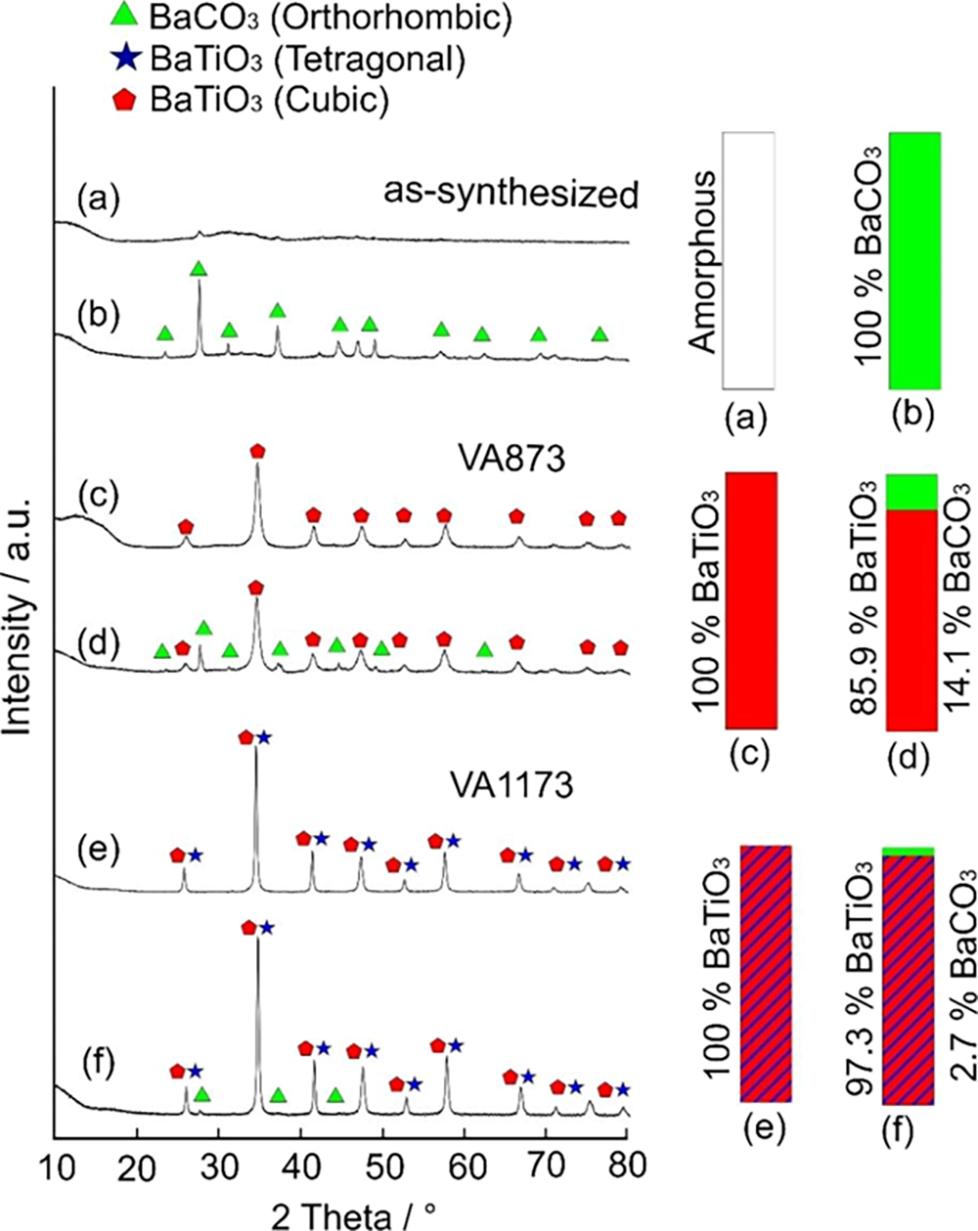
X-ray diffraction patterns and results from phase analysis of different BTO nanoparticle powders prior to (a, c, e) and after contact with liquid water (b, d, f). (a, b) As-synthesized powder, (c, d) BTO VA873 powder, and (e, f) BTO VA1173.

After water contact of the as-synthesized amorphous BTO particle powder (Figure 5a), the diffraction pattern points to BaCO_3_ as the exclusive crystalline phase (Figure 5b). BTO VA873 (Figure 5c) and VA1173 powder samples (Figure 5e), on the other hand, show BTO-specific diffraction patterns after contact with liquid water–additional features that originate from crystalline BaCO_3_ (Figure 5d,f). Rietveld analysis indicates that approximately 14.1 or 2.7% of the VA873 and VA1173 samples convert into crystalline BaCO_3_, respectively. BTO nanoparticle exposure for 7 days to liquid water, both for as-synthesized and for BTO VA873 samples, does not change the materials conversion yield (Figure S4, Supporting Information). Densely packed rods and needles comprising flattended material surfaces (Figure S4a,b, Supporting Information) are observed for as-synthesized BTO. Related measurements on VA873 powders reveal the coexistence of both qualities, BaCO_3_ rods and highly dispersed nanoparticle agglomerates (Figure S4c,d, Supporting Information). For this sample, Rietveld analysis did not reveal further changes in the crystallographic phase composition (Figure S4c,d, Supporting Information).

**Figure 6:**
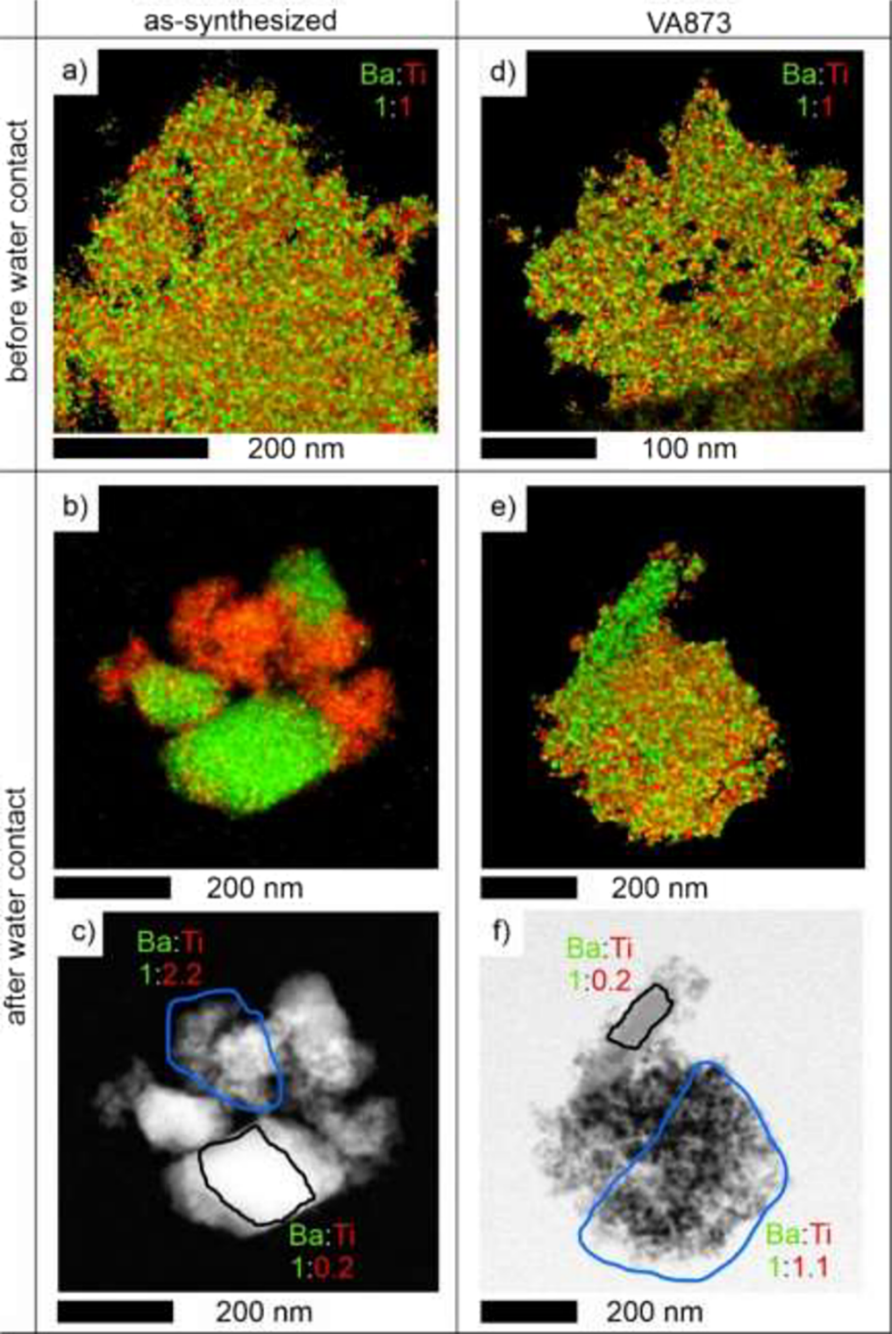
EDX quant maps acquired on as-synthesized BTO and BTO VA873 nanoparticle powders before (a, d) and after (b, e) contact with liquid water. Ba–Ti ratios of the as-synthesized and VA873 nanoparticle agglomerates before contact with water are highlighted in a and d, respectively. For water-treated samples, Ba–Ti ratios were determined for separate regions containing either predominantly nanoparticle aggregates (see blue-bordered region in c and f) or rods (black-bordered region in c and f).

Finally, we analyzed the elemental distribution of Ba and Ti in as-synthesized and VA873 samples before and after water treatment and acquired for this purpose energy-dispersive X-ray spectroscopy (EDX) maps (Figure 6, further details in Supporting Information, Figure S5).

Prior to water contact, as-synthesized BTO and BTO VA873 nanoparticle powders exhibit a homogeneous distribution of barium and titanium atoms, as shown by the EDX maps (Figure 6a,d). After data correction for software overshooting effects (see the Supporting Information for details), the Ba–Ti ratio was determined to be 1:1, which is consistent with ion concentrations of the precursor solutions and the respective flow adjusted for flame spray pyrolysis.^30,31^

Significant separation into Ba-rich regions (hosting the micrometer-sized rods) or Ba-depleted regions (at adjacent nanoparticle regions) occurs upon sample exposure to liquid water (Figures 6 and S5). For the as-synthesized BTO as well as for the VA873 sample, regions featuring micrometer-sized rods (together with some nanoparticles in their vicinity) consistently yield an estimated Ba–Ti ratio of 1:0.2. This value corroborates the assignment of the rod-shaped structures to the BaCO_3_ phase. (The non-negligible Ti signal detected for these sample spots is attributed to the presence of some BTO nanoparticles in close contact with the rods’ surface). Importantly, we found a significant difference in the elemental composition between as-synthesized BTO and the VA873 sample when analyzing the remaining nanoparticle fraction. In this case, Ba depletion is more pronounced for the as-synthesized sample (Ba–Ti = 1.0:2.2) as compared to the annealed sample (Ba–Ti = 1.0:1.1). The results corroborate the preferential Ba-leaching upon water contact from BTO nanoparticles. For the as-synthesized powders, ∼ 55% of all Ba atoms are removed upon water exposure, whereas only ∼ 9% of all Ba atoms are removed from a VA873 sample. The much stronger Ba depletion observed for the amorphous BTO powder points to barium dissolution both from the particles’ surface and from bulk regions. On the other hand, we postulate that Ba leaching from VA873 and VA1173 crystalline powders is limited to the surface of the BTO nanoparticles. In line with such an interpretation, barium leaching is less extensive for VA1173 samples, featuring a lower surface area (21 ± 3 m^2^ g^–1^) than the VA873 powder (63 ± 9 m^2^ g^–1^), thus resulting in reduced carbonate formation. While the specific surface area of the two samples (before water exposure) differs by a factor of ∼ 3, the BaCO_3_ content (as determined by phase analysis after water treatment, Figure 6) differs even by a factor of ∼ 5 (i.e., 14.1% for VA873 and 2.7% for VA1173, respectively). While these results corroborate the critical influence of the surface area on the degree of conversion, additional parameters seem to play a role.

From our observations, we deduce that at room temperature, highly dispersed BTO nanoparticle powders with specific surface areas of 60 m^2^ g^–1^ and larger are unstable in aqueous environments and transform into crystalline BaCO_3_ with characteristic crystal morphologies. The source of carbon is not perfectly clear since fractions of carbon may also originate from synthesis-related impurities as they exist on as-synthesized BTO nanoparticle powders (Figure 3e). Annealed and oxygen-treated BTO VA873 powder samples, however, are judged from thermogravimetric analysis, exempt from carbonaceous contaminants but do also convert into BaCO_3_ upon reaction with CO_2_ though to a less extent. Due to the complex interfacial materials situation related to the powder that is covered with condensed water, which in turn becomes continuously removed by drying in CO_2_-containing air, it is impossible to decide for the here-reported experiments whether the CO_2_ predominantly stems from the gas phase or serves in the form of dissolved species in liquid water as a precursor educt.

On average, cystalline BTO VA873 nanoparticles are slightly larger than amorphous as-synthesized particles. Values for the corresponding crystallite domain size (12 ± 1 nm) are consistent with the average particle size that was determined from TEM image analysis and particle counting (*d* = 17 nm, Table 3).^35^ The solid’s crystallinity or its absence has a strong impact on the dissolution behavior. It is well-established that due to the generally larger enthalpy associated with the amorphous form, both the corresponding intrinsic dissolution rate and its solubility are generally larger for amorphous solids than those for their crystalline counterparts.^36^ This is clearly reflected by the observations described in Figures 2, 4a–d, and 5. Amorphous BTO transforms into crystalline BaCO_3_ to a significantly larger extent, whereas only a fraction of 14% of the crystalline BTO seems to convert into BaCO_3_.(Figure 5c–d) Specific surface area values play an important role as well. Powders of larger and polycrystalline BTO VA1173 particles with specific surface areas around 21 m^2^ g^–1^, which corresponds to roughly a third of the BTO VA873 powder, remain essentially stable in condensed water (Figure 5e,f). Water contact leads to only 3% of the entire sample that is made up of BaCO_3_.

Apart from the general instability of anhydrous BTO nanoparticles in condensed water, it is also the enhanced kinetics of Ba extraction from the solid into the solution that represents a challenge in the liquid phase processing of BTO nanomaterials. This may be particularly relevant for the production of BTO-based electroceramics, which involves sintering as the key process step after shaping the workable mass (powder or an aqueous paste). Our literature research revealed that there exist several reports about abnormal grain growth of BTO nanomaterials after processing and sintering at temperatures *T* > 1100 °C.^37−39^ One study even described abnormal grain growth even within electrospun fibers, where water was also inevitably involved in the preceding materials processing. The authors clearly indicate that an intermediate BaCO_3_ phase has formed, which is also consistent with independent report,^40^ but decomposes at temperatures above 700 °C. In complex metal oxides and perovskites such as BTO, abnormal grain growth is a common phenomenon and can have multiple reasons.^41,42^ One obvious reason that is associated with the use of nanomaterials, however, would be that during the overall process large-grained BaCO_3_ structures of the type described in Figures 1 and 2a have formed as intermediates to serve as precursor grain for the significantly larger BTO grains that result from subsequent sintering-induced carbonate decomposition. Inspection of the electron microscopy data reported in refs (39 and 40) with structures reported in this study may substantiate this hypothesis, which is also in line with the earlier reported effects of residual BaCO_3_ on the dedensification of BTO during sintering.^43^

## Conclusions

If BaTiO_3_ nanoparticle powders are grown in the gas phase, i.e., under anhydrous conditions and in the absence of organic solvents, they exhibit enhanced reactivity toward liquid water. In many cases, however, water cannot be excluded from subsequent materials processing steps for economic and environmental reasons. This work reveals the role of size and crystallinity of highly dispersed BaTiO_3_ particle systems, which represent a prototypical nanomaterial component within ferro- and piezoelectric devices. Pronounced crystallinity and/or contact with hydroxyl-containing polymers such as poly(vinyl alcohol) (PVA) stabilizes the material against incongruent Ba^2+^ dissolution, Ba^2+^ leaching and BaCO_3_ formation. The thermodynamic instability of BTO in water and resulting deviations from the desired stoichiometry enables exaggerated grain growth and explains the sometimes observed evolution of inhomogeneous microstructures.^17,18,44^

From another perspective, it should be emphasized that the spontaneous evolution of micrometer-sized and crystalline objects such as the BaCO_3_ fibers, needles, and whiskers occurs wherever condensed water and gaseous CO_2_ come in simultaneous contact with dissolved and locally released Barium ions. In nature, cycles of carbonate dissolution and crystallization strongly impact the CO_2_ balance in the earth’s atmosphere. In particular, the reactivity of Ba^2+^ containing multinary metal oxides, e.g., in the form of waste multilayer ceramic capacitors (MLCCs), and the concomitant formation of carbonates with their characteristic crystal growth modes may be relevant for carbon capture and storage (CCS).^4^ CO_2_ binding capacity and carbonate formation of Ba^2+^ seem to be comparable to those of Ca^2+^. Control over the size of the metal carbonate particles is a key point for optimization of this CCS approach and involves parameters such as the release of Ba^2+^ ions and their local concentration in solution. This opens an opportunity window for carbon capture in combination with the spatially controlled deposition of carbonate structures at natural and engineered surfaces and pore structures.

## Supporting Information Available

Additional transmission electron microscopy data and electron diffraction data of electrospun metal oxide and carbonate particles and EDX quant maps showing the elemental distribution of Ba and Ti ions (PDF)
