# Supplementary material for: Water-Mediated Conversion of BaTiO3 Nanoparticles into BaCO3 Nanorods in Electrospun Polymer Fibers: Implications for Carbon Capture Applications
Source: ACS Appl Nano Mater. 2023 Oct 19;6(21):19887–95. doi: 10.1021/acsanm.3c03703 (PMC10644300; doi:10.1021/acsanm.3c03703)
Supplement: Supplementary file 1 — an3c03703_si_001.pdf [file an3c03703_si_001.pdf]

Supporting Information

$BaCO_3$ Formation in Aqueous $BaTiO_3$ Particle Systems:

A Nanomaterials Processing Topic

Hasan Razouq[1], *Kerstin Neuhauser*[1], *Gregor Zickler*[1],

Thomas Berger[1], *Oliver Diwald*[1*]

[1]Department of Chemistry and Physics of Materials, Paris-Lodron University Salzburg,

Jakob-Haringer-Straße 2a, A-5020 Salzburg, Austria

E-mail: oliver.diwald@plus.ac.at

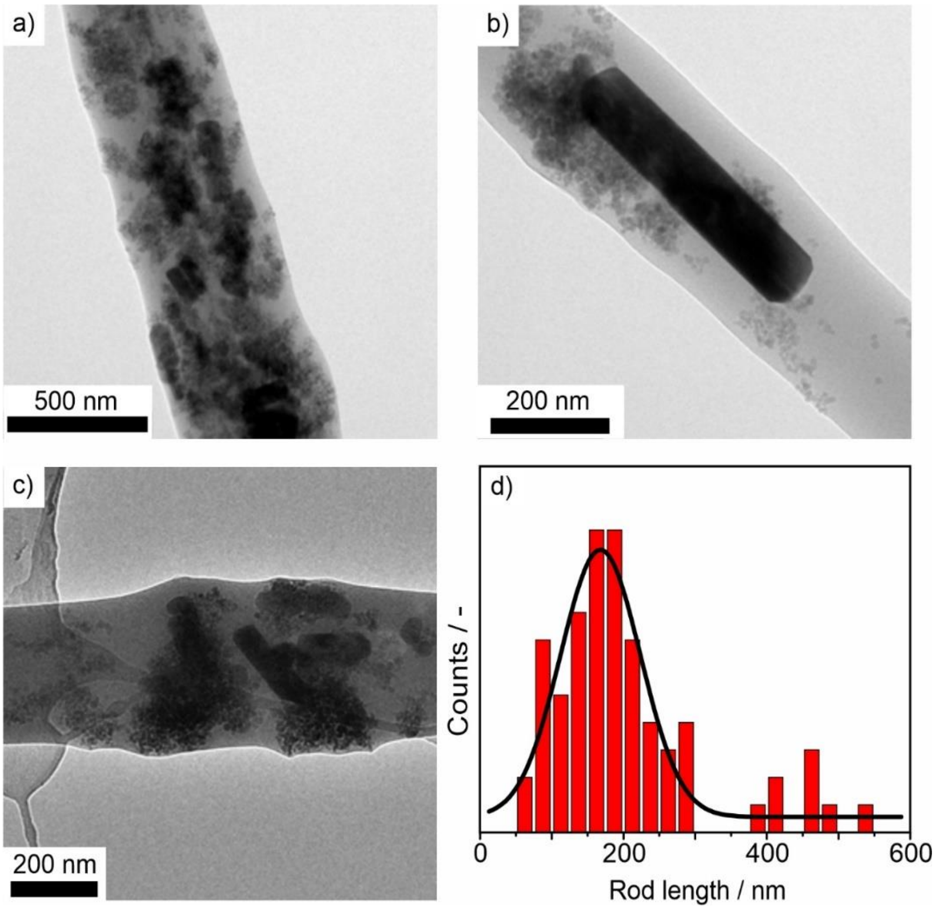

Figure S1. TEM images of as-synthesized BTO nanoparticles inside electrospun PVA fibers (a, b and c) and length distribution plot of the elongated rod-like features inside the PVA fibers (d).

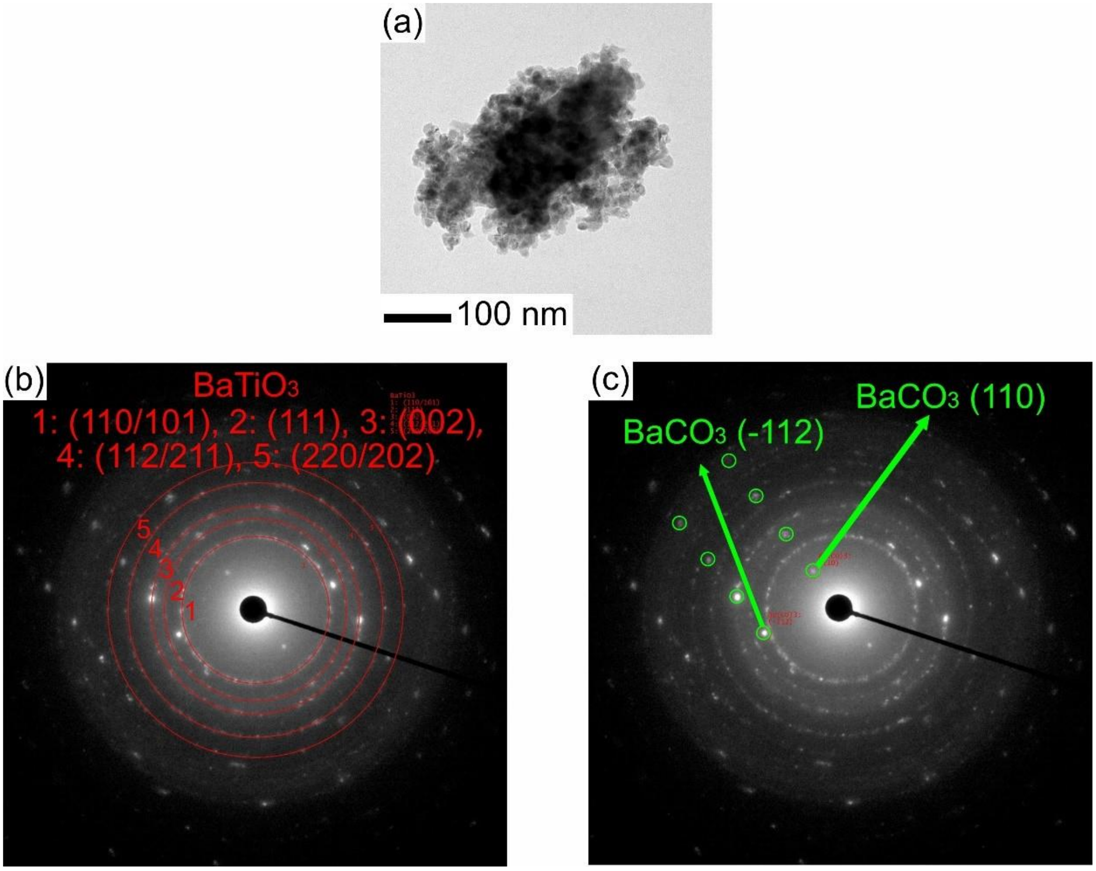

Figure S2. TEM image (a) and selected area electron diffraction (SAED) patterns (b, c) of a VA873 nanoparticle powder agglomerate after contact with liquid water and subsequent drying. Diffraction spots reveal the presence of crystalline BTO (red circles in b) and crystalline barium carbonate (green spots in c). Many small BTO crystallites with random crystal orientation contribute to the diffraction feature in (b). On the other hand, the diffraction spots corresponding to the barium carbonate phase (green circles in c) result from only two large single crystals oriented in the same direction in the selected area (a).

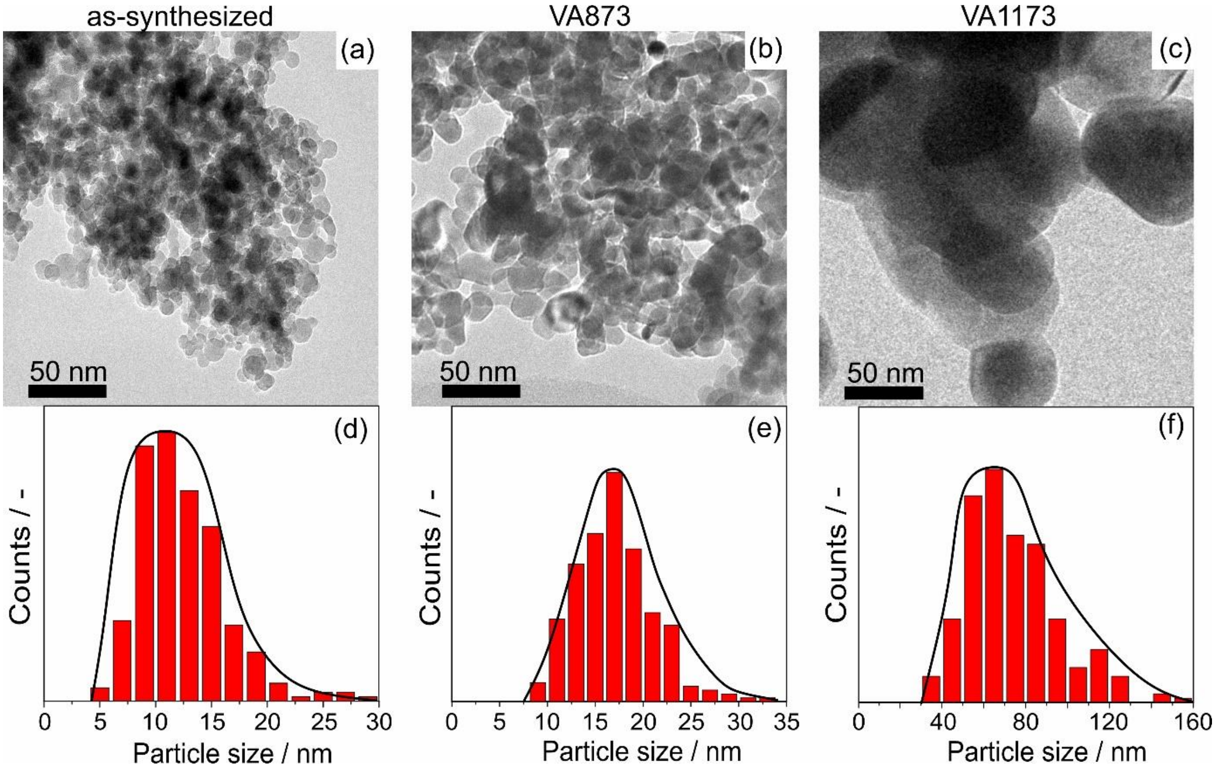

Figure S3. TEM images (a, b, c) and particle size distribution plots (d, e, f) of as-synthesized (a, d), and annealed BTO nanoparticle powders: BTO VA873 powder (b, e) and BTO VA1173 (e, f).

The as-synthesized powder is characterized by a narrow particle size distribution, in the range below 30 nm and with an average size of 13 nm. Despite a slight growth of the average particle size from 13 to 17 nm, these characteristics are essentially retained after annealing to 873 K. Thermal annealing to 1173 K induces substantial particle coarsening and leads to a broader particle size distribution with a maximum at 73 nm.

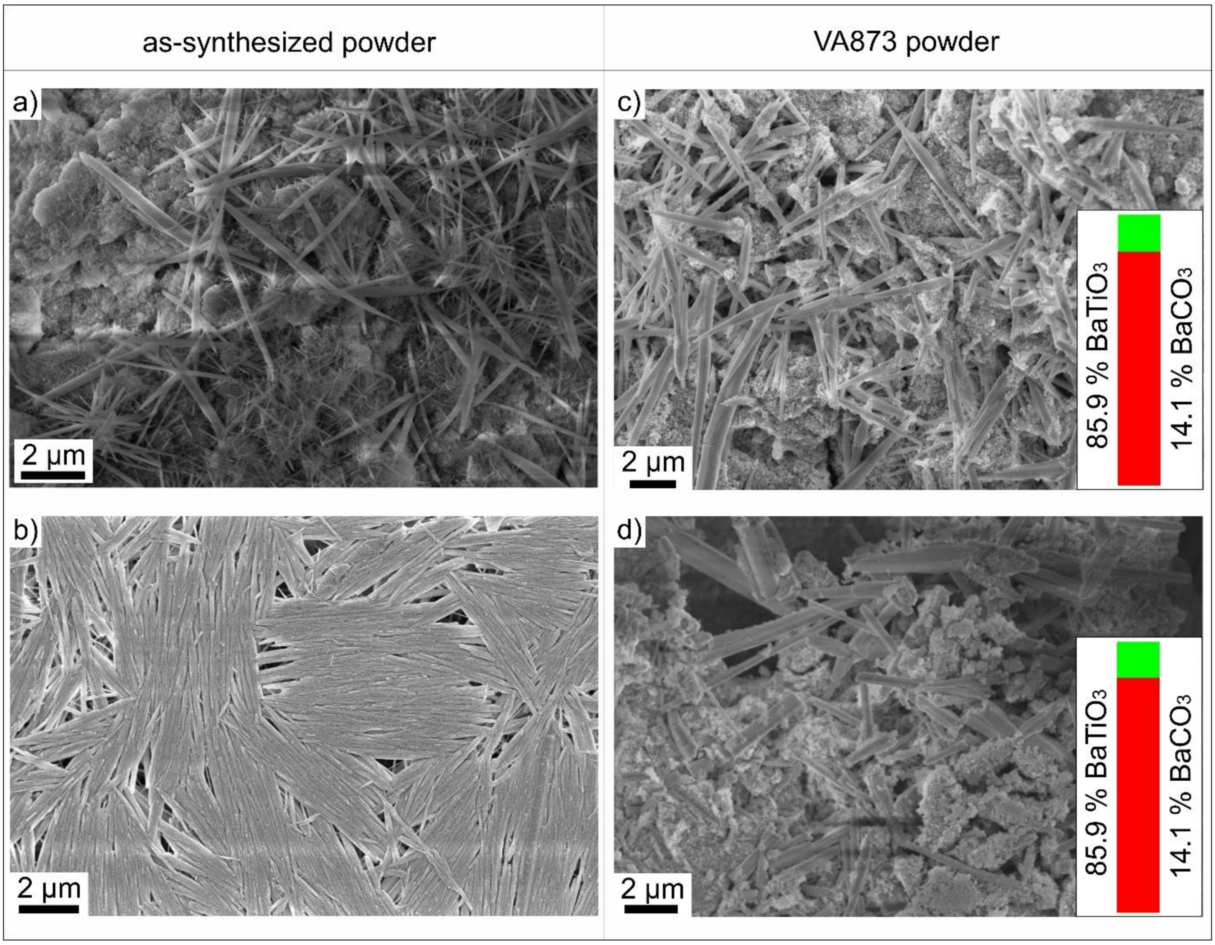

Figure S4. SEM images of as-synthesized BTO (a, b) and of BTO VA873 nanoparticle powders (c, d) after the exposure of 50 mg of powder to a single water drop (a, c) and after one week of water contact (b, d). Samples were dried at 80 °C in air prior to microscopic analysis. Insets in (c) and (d) highlight the abundance of the observed crystalline phases as determined by Rietveld refinement of XRD data.

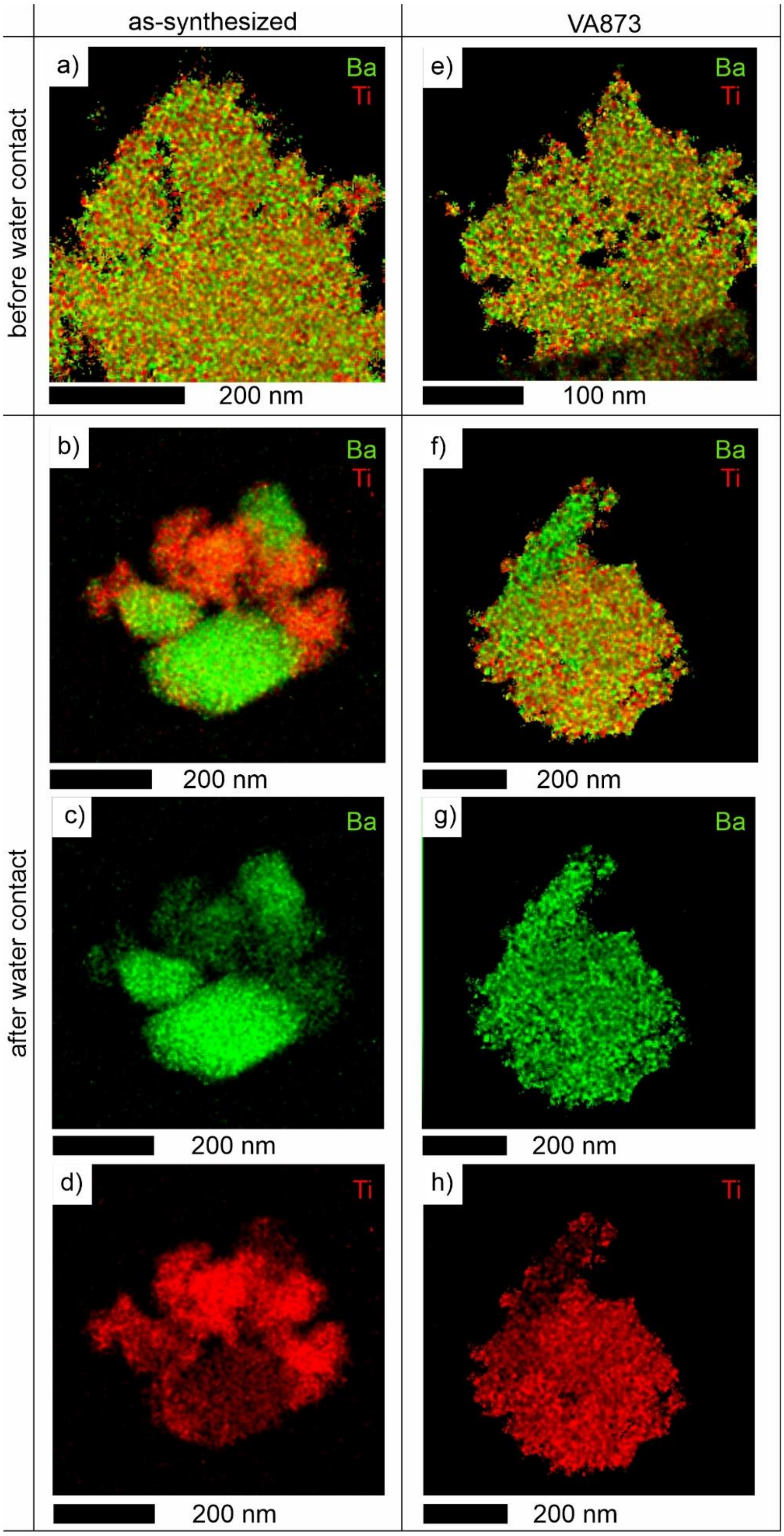
as-synthesized
VA873
before water contact
after water contact
a)
Ba
Ti
200 nm
e)
Ba
Ti
100 nm
b)
Ba
Ti
200 nm
f)
Ba
Ti
200 nm
c)
Ba
200 nm
g)
Ba
200 nm
d)
Ti
200 nm
h)
Ti
200 nm

Figure S5. Mixed (Ba, Ti) EDX quant maps acquired on as-synthesized BTO nanoparticle powders (a, b) and on a VA873 sample (e, f) before (a, e) and after contact with liquid water (b, f). Single elemental maps of Ba (c, g) and Ti (d, h) are shown in addition to the mixed elemental maps (b, f) for the water treated samples.

Correction of software overshooting:

The Ba:Ti ratio as determined from EDX raw data is 1.0:1.4 for both powder samples before contact with water. On the other hand, the X-ray diffractogram of the VA873 powder showed exclusively BTO diffraction features. The deviation from the expected Ba:Ti ratio of 1:1 results from software overshooting of the Ti quantity due to an overlap of Ti and Ba peaks (resulting from a Lα energy of 4.465 keV for Ba and of 4.510 keV for Ti). A correction factor was thus applied to all Ti raw data to account for this effect.
